# Lessons learned from an unsuccessful decentralized clinical trial in Oncology

**DOI:** 10.1038/s41746-024-01214-5

**Published:** 2024-08-13

**Authors:** Antonis Valachis, Henrik Lindman

**Affiliations:** 1 Department of Oncology, Faculty of Medicine and Health, Örebro University Hospital, Örebro University, Örebro, Sweden; 2 Department of Immunology, Genetics and Pathology, Experimental and Clinical Oncology; Clinical Oncology, Faculty of Medicine, Uppsala University Hospital, Uppsala, Sweden

**Keywords:** Clinical trial design, Cancer

## Abstract

Decentralized clinical trials have gained in popularity over the last years due to their advantages related to broadening recruitment strategies and resource saving possibilities. As more clinical trials adopt decentralized strategies, it is essential to share the knowledge about both successful and unsuccessful efforts in the research community. In the present commentary, we explore potential reasons that led to early termination of a decentralized clinical trial in Oncology.

## Decentralized clinical trials and the paradigm of TELEPIK trial

Decentralized clinical trials (DCTs) refer to trials where study-related processes, including data collection during study that would historically have only been carried out at a trial site, are performed remotely using technological means or other remote services instead of more traditional approaches^1,2^. DCTs may be fully decentralized, where no research processes occur at a research site, or more commonly hybrid trials where some processes occur remotely, and others onsite. DCTs have several advantages compared to traditional clinical trials and are expected to be further optimized and implemented in clinical research during the upcoming years^1,2^. The main advantages of DCTs rely on the possibility for a broader patient inclusion since even patients living in rural areas without easy access to clinical research centers or patients from lower socioeconomic backgrounds can be included in clinical trials. Additionally, replacing on-site visits with remote ones is not only less costly for sponsors but also reduces participants’ burden^1–3^.

Recognizing the potential advantages of DCTs, the TELEPIK trial was designed aiming to provide proof-of-concept regarding remote follow-up strategies for adverse events in cancer patients treated with targeted therapies^4^. In TELEPIK trial, patients with advanced estrogen-receptor (ER) positive /HER2-negative breast cancer eligible for a combination of alpelisib and fulvestrant (patients with activating somatic PIK3CA-mutation fulfilling eligibility criteria similar to SOLAR-1 trial^5^) were eligible to be included and be followed remotely through a dedicated cloud-based, patient-centric mobile platform provided by a vendor. The spectrum of adverse events that can be expected due to alpelisib as hyperglycemia, skin rash, and diarrhea^6^ were considered as suitable adverse events to be followed through a mobile platform. In fact, the technological solution enabled the patients to share their self-monitored glucose measurements, to inform the trial personnel regarding any adverse event through the mobile platform and included a secure video-conferencing platform as well to further support a remote assessment of adverse events as skin rash. Through its decentralized approach, TELEPIK would also facilitate patient inclusion by enabling patients from nearby hospitals to participate through referral to the two trial sites (University Hospital of Uppsala, Örebro University Hospital, Sweden). The inclusion and exclusion criteria used in TELEPIK trial were similar to SOLAR-1 trial with the addition of criteria related to patients’ ability to operate a smartphone compatible with telemedicine platform. TELEPIK trial offered the possibility of utilizing a new targeted therapy, namely alpelisib, after disease progression on CDK 4/6-inhibitors which was at that time not allowed according to restrictions on reimbursement policy in Sweden that recommended alpelisib only after progression on endocrine therapy as monotherapy. Unfortunately, the TELEPIK trial was terminated early due to low accrual since only two patients were included in an inclusion period of 6 months whereas the target accrual was 20 patients^4^.

The early termination of TELEPIK trial due to low accrual contrasts with what it is expected from a DCT where one of the advantages is the possibility to include more patients by expanding the potentially eligible patient cohort. In fact, an enrollment rate of 8% (2 enrolled patients out of 25 estimated eligible patients) for both trial sites and nearby hospitals that would refer potentially eligible patients was observed that is similar to the anticipated rate in traditional oncology trials^5^. So why did the TELEPIK trial not achieve its goal?

## Potential reasons for early termination of TELEPIK trial

In an effort to better understand the reasons why TELEPIK trial did not achieve its scope, we asked trial personnel (investigators in trials sites, study nurses) as well as breast oncologists from nearby hospitals their view on TELEPIK trial design through five questions that were chosen based on primary investigators’ understanding on potential reasons for early termination: how did the decentralized approach affect your ability to recruit patients; how did the requirement for patients to be referred to another hospital affect the recruitment possibilities; did you perceive, based on the trial protocol, that you would lose “control” over your patients if they were included in the study; what difficulties did you experience in finding patients for the study; how did the fact that there would be fewer visits to the hospital impact the recruitment possibilities.

In this commentary, we will focus on aspects related to patient recruitment strategies including technological barriers and referrals within a DCT because these aspects can potentially be captured by the questions posed to trial personnel.

The technological solution that was used in the TELEPIK trial was based on an external system that was outside of the hospital-based infrastructure. The use of technological means that are not integrated in the hospital-based infrastructure can be a barrier for the successful conduction of a DCT. The utilization of different technological solutions for trial conduction is a common practice in traditional clinical trials as well but it can be exaggerated in DCTs. In fact, the advantages of DCT in terms of remote monitoring and follow-up can be diminished by the complexity of using new systems that are not integrated in the technological solutions that are used in clinical practice by the hospitals. This is related to a hesitation of using multiple technological solutions that is evident among clinicians^7,8^. As a result, the positive attitude among physicians towards using technological solutions can be negatively influenced by the complexity of using different solutions^7^. The utilization of patient-directed remote services as a part of DCTs has several advantages but it could also be a barrier if the technological solutions are complex or have poor help desk services for participants or study personnel. The latter was a point that was raised from study nurses involved in TELEPIK trial where help desk was difficult to reach from both study personnel and patients.

The lack of integration between technological solutions used in DCTs and hospital-based infrastructure might also have implications in the resources that are needed for study completion. DCTs are considered cost-saving by reducing the number of onsite patient visits and decreasing the costs related to time for study nurses and clinicians^3^. However, learning and using a new technological solution that is completely different from the solutions used in clinical practice and outside the hospital infrastructure can be time-consuming for study nurses and investigators thus resulting in “digital overload”, increasing the resources needed and jeopardizing the potential benefit of DCTs in terms of cost-saving^9^.

Another potential barrier when implementing DCTs using nearby hospitals for patient referrals should be highlighted. It is beyond doubt that DCTs enable a broader patient inclusion since patients do not need to travel to the trial site, but they can participate remotely. However, patient referrals from nearby hospitals can be complicated by physician- or patient-related factors. From a physician’s perspective, having a patient in a DCT outside the hospital might pose challenges on how the responsibilities between the investigator and the treating physician are divided and can lead to uncertainties that might influence the willingness of treating physician to refer a patient. This point was raised by breast oncologists from nearby hospitals in the example of TELEPIK trial as an important aspect to be considered during the design of DCT. This issue is more relevant in referrals for enrollment in DCTs where enrolled patients often continue their follow-up through their hospitals compared to referrals for enrollment in traditional trials where enrolled patients are followed in the study site where they have been enrolled. From a patient perspective and recognizing the important role of physician–patient relationship to patient’s well-being^10^, a referral to another hospital for participating in a DCT where less onsite patient visits are planned can be considered as a less personal-oriented healthcare. This potential obstacle is easier to overcome in referrals for inclusion to traditional trials since they often include frequent physical patient visits thus enabling a patient–physician relationship to be established whereas it might be more difficult in DCT where physical patient visits are limited. This potential lack of trust can impact patients’ satisfaction with medical care^11^ but also patients’ willingness to participate to a clinical trial^12^.

## Lessons learned from the TELEPIK trial

Identifying the issues that led to the early termination of a clinical trial can help investigators and stakeholders understand potential barriers and develop suitable mitigating strategies. This is of particular importance for DCTs due to the lack of evidence comparing the performance of this study design with conventional clinical trials in terms of recruitment, retention, and cost^13^. After the termination of the TELEPIK trial, fruitful discussions between the sponsor and the investigators were performed to identify potential reasons that led to early termination. Additionally, trial personnel in study sites and breast oncologists from nearby hospitals were included in the discussions to gain more insights on their perception on the TELEPIK trial as DCT. What lessons have we learned from TELEPIK regarding the design and conduct of DCTs? In our opinion, using technological solutions that are not integrated to hospital infrastructures could be a barrier impacting the successful conduction of a DCT. Furthermore, the utilization of nearby hospitals for referral should be accompanied by a clear description of investigator-related and treating physician-related responsibilities and should also be discussed with the patients to avoid misconceptions that can influence the patient–physician relationship and become a barrier for study inclusion. It is important to point out that TELEPIK recruitment strategy did not utilize any digital recruitment approaches, but the strategy was conventional in terms of capturing patients through trial sites and referrals from nearby hospitals from a research network. This was in line with TELEPIK trial design where some patient visits were obligated to be physical, thus the trial was not fully decentralized. Whether digital recruitment strategies would increase enrollment rates cannot be answered through the TELEPIK trial example.

The advantages of DCTs in the current era of technological advances are beyond doubt but the methodological approaches for study design and conduction can be optimized. Promotion of a more equal representation of study participants, facilitation and enhancement of data collection process, and reduction of patients’ burden and expenses associated with clinical trial procedures are some of the main advantages of DCTs that TELEPIK trial also utilize during trial design and execution. However, the lessons learned from early termination of the TELEPIK trial, can, hopefully, give some insights to clinical researchers and stakeholders on how to optimize the design and conduction of DCTs and limit the risk for publication bias regarding DCTs^13,14^.
